# ‘We view that as contraceptive failure’: Containing the ‘multiplicity’ of contraception and abortion within Scottish reproductive healthcare

**DOI:** 10.1016/j.socscimed.2012.12.004

**Published:** 2013-03

**Authors:** Siân M. Beynon-Jones

**Affiliations:** Science and Technology Studies Unit (SATSU), Department of Sociology, University of York, York, YO10 5DD, UK

**Keywords:** Scotland, Abortion, Contraception, STS, Gender, Fertility prevention

## Abstract

Within contemporary Scottish policy guidance, abortion is routinely configured as evidence of a resolvable problem with the healthcare provision of contraception. This article draws on 42 semi-structured interviews with Scottish health professionals conducted during 2007–2008, in order to explore how, and in what form, realities of contraception/abortion are sustained within abortion practice. In addition to providing empirical insights concerning this sociologically neglected aspect of reproductive healthcare, it demonstrates how a novel conceptual approach could be used to develop existing social scientific analyses of the provision of techniques of fertility prevention. Science and Technology Studies (STS) has highlighted the importance of studying the complex socio-material practices through which realities are enacted (or ‘performed’). Mobilising this insight, my analysis illustrates the complex socio-material work required to enact abortion as evidence of a ‘problem’ with contraception that is resolvable within the healthcare consultation. This work, I argue, renders visible the ontologically ‘multiple’ (Mol, 2002) nature of contraception/abortion, with important implications for both social science and policy approaches to these techniques of fertility prevention.

## Highlights

► Analysis of Scottish health professionals' accounts of providing contraceptive advice during consultations about abortion. ► Draws on STS theory to conceptualise ‘contraception’ and ‘abortion’ as realities enacted through practice. ► Explores how contraception/abortion realities are enacted through Scottish abortion consultations. ► Illustrates the work involved in aligning these realities with the contraception/abortion enacted through policy.

## Introduction

Policy guidance concerning sexual health services in Scotland positions the provision of contraceptive advice during consultations about abortion as a means through which to reduce the rates of the latter:

Approximately 1 in 4 women who have a termination of pregnancy subsequently have another termination of pregnancy. Advice about effective contraception following termination of pregnancy is essential to reduce termination of pregnancy rates. (NHS Quality Improvement Scotland, 2008, p. 15)

Accordingly, recent standards for Scottish abortion services require explicitly that:

60% of women leave the facility with one of the more effective methods of contraception (hormonal oral contraceptives, intrauterine devices or contraceptive implants). (NHS Quality Improvement Scotland, 2008, p. 15)

Similar framings of the relationship between contraception, abortion and reproductive healthcare are also prevalent in UK sexual health guidance more broadly (for example, Department of Health, 2009; Medical Foundation for AIDS & Sexual Health (MedFASH), 2008; National Collaborating Centre for Women's and Children's Health, 2005). A particularly notable example is the recent guidance commissioned by NICE, which depicts the provision of Long Acting Reversible Contraception (LARC) to women as a revolutionary means to reduce the UK's abortion rate (National Collaborating Centre for Women's and Children's Health, 2005).

In this paper I use Scottish health professionals' accounts of the provision of contraceptive advice during consultations about abortion to explore some of the implications of these forms of guidance. My analysis of health professionals' accounts illustrates the socio-material work that is necessary in order for abortion to be enacted as evidence of a ‘fixable’ problem with contraception. In illustrating this phenomenon, I have two aims. Firstly, to explore a neglected empirical topic, namely, the provision of techniques of fertility prevention within contemporary Scottish reproductive healthcare. Secondly, to suggest new theoretical directions for broader social scientific enquiry concerning the provision and use of techniques of fertility prevention.

## Background: the differentiation of abortion and contraception through twentieth century medicalization in the UK

For much of the twentieth century, *all* techniques of fertility prevention were deemed illegitimate by the UK medical profession and, more broadly, within public discourse (Brookes, 1988; Hawkes, 1996; McLaren, 1990; Thomas, 1985). As McLaren (1990) demonstrates, the gradual differentiation of contraception and abortion as two distinct techniques was the result of a strategy employed by the early twentieth century birth control movement. In attempt to gain the support of the medical profession, birth control campaigners promoted the use of relatively ‘high-tech’ pre-coital methods (for example, the diaphragm and cap, and later, hormonal contraceptives and intrauterine devices) whose use could be planned far in advance of sex, and required clinical supervision. In contrast, they explicitly dissociated themselves from methods associated with (hetero)sex, or its aftermath (condoms, ‘withdrawal’ and abortion).

In spite of the campaigns of birth control activists, their advocacy of the idea that fertility should be ‘planned’ through the use of pre-coital contraception did not become accepted as a mainstream part of medical practice until the 1960s–70s (McLaren, 1990). McLaren suggests that the availability of a more high-status and ‘scientific’ (Clarke, 1998) method of contraception in the form of the Pill was critical in the profession's change of heart concerning the goals of the birth control movement. Another important event was that, during the late 1960s, health professionals in much of the UK suddenly became faced with responsibility for the provision of abortion (Aitken-Swann, 1977; Davis & Davidson, 2005; Hawkes, 1996; McLaren, 1990; Thomas, 1985). The 1967 Abortion Act re-classified abortion as a ‘medical’, rather than a ‘criminal’ act (Sheldon, 1997) by defining legal grounds on which two doctors could agree that it was necessary in the interests of a pregnant woman's health, or the health of her foetus. In doing so, it gave doctors formal responsibility for the decision about whether or not a particular pregnancy should be terminated (Davis & Davidson, 2005).

The legislative framework introduced by the 1967 Abortion Act continues to regulate the provision of abortion in the UK (with the notable exception of Northern Ireland, to which this Act has never been applied, and where abortion remains largely unavailable). While it has, arguably, facilitated the widespread provision of safe, legal procedures (Sheldon, 1997) the law nonetheless legitimates abortion only as a last resort course of action necessary to alleviate the suffering of a patient whose unwanted pregnancy constitutes a threat to her mental and/or physical wellbeing (Boyle, 1997; Sheldon, 1997). Negative framings of abortion clearly influenced the incorporation of contraceptive provision into UK healthcare; this was positioned as necessary ‘to prevent illegitimacy and abortion rather than to promote sexual freedom’ (Thomas, 1985, p. 52). While illegitimacy is no longer an explicit policy concern, the guidance cited above demonstrates that concerns about the prevention of abortion remain significant.

## Conceptual framework

The significance of professional practice concerning the provision of contraceptive advice to women seeking abortion was first highlighted by Luker (1975) in her ground-breaking study of contraceptive ‘risk taking’. In an exploration of the non-use of contraception by women who request abortion, Luker argued that competing perceptions of the meanings of contraception and unwanted pregnancy/abortion are held by women and medical institutions. She suggested that the latter assume that the most significant ‘cost’ of (hetero)sex is ‘unplanned’ pregnancy/abortion. Simultaneously, medical institutions minimise the costs of contraception, and stigmatise women who refuse to bear these costs as either ‘ignorant’ or ‘self-destructive and irrational’ (Luker, 1975, p. 140).

The central aim of Luker's study was to destabilise these assumptions by illustrating that, when contraception is situated within the lived context of its use, its non-use becomes an entirely rational act. She argues that:

risk-taking behaviour which ends in an unwanted pregnancy is the result of a “rational” decision-making chain produced by a person who is acting in what he or she perceives to be his or her best interests, although often in the presence of faulty data. (Luker, 1975, p. 138)

Costs of contraception can include, for example, the side-effects of hormonally-based contraceptives, the routine interactions with clinicians that these drugs necessitate, and costs to identities and relationships. In particular, as Luker points out, to obtain and make use of a contraceptive involves the cost of acknowledging to oneself and others (often health professionals) that one is *planning* to be sexually active. In contrast to these immediate costs of contraception, ‘unplanned’ pregnancy/abortion represents an unknown future cost which may be ‘discounted’, or may in some cases be viewed as a benefit, for example, an opportunity to test a male partner's commitment (Luker, 1975).

However, as Paxson (2004) highlights, while Luker's work provides valuable insights, it replicates an important aspect of the institutional discourse which it sets out to critique. As numerous commentators have noted (Ali, 2002; Paxson, 2004; Ruhl, 2002) the medicalization of techniques of fertility control is grounded in a socially specific construction of human subjectivity. Specifically, it reflects Western Enlightenment philosophy's account of subjectivity as contingent upon an individual's ability to abstract themselves from ‘time, space, and bodily circumstances’ (Ruhl, 2002, p. 644) in order to make rational-calculative decisions that maximise self-interest. In the case of techniques of fertility control, medical institutions view self-interest as maximised when women have control over the timing of conception. While Luker successfully illustrates that this is not the only way in which women can realise their interests, she nonetheless portrays the autonomous, rational, calculation of self-interest as the basis for women's contraceptive (non)use. In other words, she concurs with institutional logics concerning the forms of human agency which it is possible to exert in relation to techniques of fertility control (Paxson, 2004).

In contrast to Luker's analysis, anthropological studies have instead sought to illustrate how cultural norms (in particular, those concerning sexuality and fertility) shape the forms of agency which people exert through their engagement with techniques of fertility prevention. For example, Paxson (2004) demonstrates that, in Greece, women's use of techniques of fertility prevention has historically been oriented towards the maintenance of gender norms concerning masculine dominance/feminine passivity in sexual relations. Women-controlled methods of contraception, which require women to be pro-active in advance of sex, challenge these relationships. In contrast, abortion provides a private, post-hoc means for women to regulate their fertility, which does not impinge upon the norms of heterosexual encounters. Paxson argues that, within the Greek context, the introduction of medicalized models of fertility prevention in the form of ‘family planning’ initiatives can be understood as burdening, rather than liberating, women. Such initiatives stigmatise Greek women's use of abortion and require them to ‘plan’ contraception, without acknowledging that contraceptive planning *also* produces stigma because it requires women to transgress local gender norms (Paxson, 2004).

While studies such as Paxson's raise important questions about the forms of human subjectivity facilitated by the medicalization of techniques of fertility prevention (see also Ali, 2002), they nevertheless contain an important limitation. Specifically, they explore how human subjectivities are negotiated in relation to techniques of fertility prevention without considering how such techniques are themselves involved with, and (re)constituted by, practices of human meaning-making.

Other studies have emphasised the way in which human practices shape the meanings of techniques of fertility prevention (Barrett & Harper, 2000; Georges, 1996; Renne, 1997; Russell, Sobo, & Thompson, 2000; Simonds & Ellertson, 2004; Ziebland, 1999). For example, Ziebland (1999) illustrates how the medicalized framing of contraception as a technique to be planned and used in advance of sexual activity leads UK health professionals to demarcate emergency contraception (a technique used up to 72 h after intercourse) as distinct and problematic in relation to other contraceptives. However, even in studies such as this, techniques of fertility prevention are explored only in terms of the ways that they are *conceptually* shaped by social norms. There is no consideration of the ways in which the *materiality* of these techniques may be shaped by, or indeed may be involved in, practices of human meaning-making.

This omission reflects a broader pattern in existing social scientific literature concerning the provision of techniques of fertility prevention, namely, the bracketing of the nonhuman world as uninvolved in, and unaffected by, human action. This phenomenon is captured in the introduction to a key text on the subject, where Russell et al. (2000) justify the anthropological analysis of contraception on the grounds that ‘contraceptives are […] social conceptions *as well as* physical facts’ (p. 6 – emphasis added). As feminist Science and Technology Studies (STS) theorists have long argued (Haraway, 1991; Roberts, 2007) the portrayal of the nonhuman/physical world as uninvolved in practices of human (social) interpretation is precisely what allows science to claim a position of ‘objectivity’ in relation to the nonhuman world, i.e. the authority to represent this world as it ‘really’ is. By replicating science's positioning of the nonhuman world, existing social scientific literature thus re-asserts the authority of medical institutions to describe the ‘reality’ of techniques of fertility prevention.

In this paper, I suggest that STS theory provides a useful means of circumnavigating this problem. As Law (2008) notes, while STS comprises several (often competing) theoretical strands, a crucial analytical move which unites a great deal of its theory is the de-centring of human agency and the acknowledgement of the liveliness (Haraway, 1991) of the nonhuman world. Crucially, in creating space for nonhumans (or ‘materiality’), STS does not replicate scientific discourse by claiming social scientific access to the ‘reality’ of such entities. Rather, its concern is to explore how realities are ‘achieved’ or *enacted* (Mol, 2002, pp. 32–33) through the inextricable, and always re-negotiable, socio-material interactions of humans and nonhumans.

A key illustration of this approach within the field of healthcare is provided by Mol's (2002) ethnography of a single disease – atherosclerosis – which she finds to be ‘multiple’ through her exploration of its enactment across different sites within the same hospital. For example, the atherosclerosis enacted through diagnostic consultations with patients (where interview questions are used to determine precisely how much pain patients experience in their legs when walking) is not the same entity as the atherosclerosis enacted through the practices of the pathology lab (where microscopes are used to determine the degree of vessel wall thickening in stained cross-sections of excised leg arteries). In spite of this multiplicity, however, Mol insists that the reality of atherosclerosis does not become plural: a single disease named ‘atherosclerosis’ continues to be diagnosed and treated across different sites of practice. This broader process, she suggests, itself relies on particular sets of socio-material practices through which atherosclerosis's multiplicity is ‘co-ordinated’ (Mol, 2002, pp. 53–85) in such a way that it can be treated.

The central point illustrated by Mol's (2002) case study is that ‘the real is relationally enacted in [socio-material] practices,’ and that ‘if those practices were to change *the real would also be done differently*’ (Law, 2008, p. 635 – emphasis in original). Through the analysis that follows, I aim to illustrate what social scientists might gain by bringing STS's focus on socio-material practices to the study of the provision of techniques of fertility prevention.

## Methods

The findings presented here are drawn from 42 semi-structured interviews that I conducted in 2007–2008 with Scottish health professionals concerning their involvement in abortion practice in the absence of diagnosed foetal impairment. These interviews were carried out as part of a broader study which set out to address Scottish health professionals' accounts of contemporary abortion practice (Beynon-Jones, 2012, in press). The rationale for researching this group of professionals was, firstly, the important position they occupy as gate-keepers to abortion and secondly, the absence of contemporary qualitative sociological research concerning their experiences (for earlier studies and historical analysis see Aitken-Swann, 1977; Allen, 1985; Davis & Davidson, 2005; Horobin, 1973; Macintyre, 1977).

In contrast to England and Wales, where a large proportion of abortions are conducted within the specialist independent sector under NHS contract, over 99% of abortions performed annually in Scotland are conducted in NHS hospitals (Information Services Division Scotland, 2011). The absence of an independent sector in Scotland means that, in order to access abortion, women must generally be referred either by a GP or by a community sexual health clinic to the appropriate NHS hospital service. To reflect this system of provision I interviewed GPs (20), obstetrician/gynaecologists (12) and gynaecology nurses (10). A purposive approach (Ritchie, Lewis, & Elam, 2003) was used to obtain a sample that was as balanced as possible in terms of gender (15 men and 27 women) and which varied in terms of age (the overall age range of the sample was 31–61 years old) as well as the geographic/organisational location of practice (collectively, the sample was drawn from 16 different GP practices, 2 sexual health clinics and 7 hospitals). As qualitative research, the findings presented here are not intended to be representative; the numbers of health professionals interviewed reflect the point at which no new research themes were emerging during interviews.

Prior to the recruitment of participants, the study was reviewed in accordance with the University of Edinburgh's School of Social and Political Studies research ethics audit process. All interviewees had the opportunity to reflect upon a written summary of the study prior to interviews and to ask questions. Written consent was obtained from all participants. Interviews were audio recorded (except in two cases where permission was refused and where, with permission, detailed notes were instead taken), transcribed, and analysed with the aid of qualitative data management software. The analysis presented here was informed by the conceptual approach outlined above. It explored the socio-material practices through which health professionals described enacting the reality of contraception/abortion during the provision of contraceptive advice in abortion consultations.

My use of interviews as a research method through which to explore socio-material practices contains an obvious limitation. Rather than offering an indication of the socio-material practices which actually take place in the abortion clinic, interview data arguably evidence the purely *rhetorical* (social) work that is required to enact contraception/abortion in accordance with social norms (for example, those of UK healthcare policy). Such rhetorical work acquires particular significance in the context of occupations such as abortion provision, where workers routinely have to negotiate the social stigma attached to their clients' actions (Harris, Debbink, Martin, & Hassinger, 2011; Joffe, 1986; Lipp, 2011; O'Donnell, Weitz, & Freedman, 2011). Nevertheless, while it is important to remain aware of the rhetorical work which may be performed through interview accounts, it seems equally inappropriate to dismiss interviewees' descriptions of their day-to-day socio-material work practices as ‘mere’ rhetorical performances. On these grounds, in the analysis that follows I take my cue from Mol (2002, p. 27), who suggests that social scientists should try listening to health professionals ‘as if they were their own ethnographers’ of the events that have happened to them.

## Findings

Echoing healthcare policy, interviewees routinely described abortion as an indicator of a problem with contraception that could be resolved within the healthcare consultation. Crucially, however, the target of healthcare practice is very different to that of healthcare policy: the latter is concerned with overall ‘rates’ of contraception/abortion usage whereas the former is concerned with individuals' usage of contraception/abortion. In the analysis that follows I illustrate that, in describing how they negotiate the complexities generated by interacting with individual users, health professionals portray four forms of socio-material work as integral to the enactment of abortion as evidence of a ‘fixable’ problem with contraception. In drawing attention to the practices required for abortion to ‘become’ evidence of a resolvable problem with contraception, health professionals' accounts highlight the possibility of alternative practices, and hence, alternative contraceptions/abortions.

### Working on/with female subjects

Health professionals' enactment of abortion as an indicator of a ‘fixable’ problem with contraception co-constructs a particular form of female subjectivity. Specifically, this enactment of contraception/abortion requires the *naturalisation of* contraceptive control over pregnancy as an intrinsic state of ‘being’ a fertile female subject:

…we would always kind of then try and, and hospitals would usually do that too, sort of say well, ‘Obviously this was unplanned, don't want this to happen again I'm sure, need to think about contraception,’ and we view that as contraceptive failure, lack of contraception. [GP7, male]

I: Um and in the consultation as well would you discuss um contraception with women at all or?

P: Yeah I mean we would look at […] sort of both sides of it and I would usually start that part of the consultation by asking them, ‘You know at the time you got pregnant were […] you using any contraception, were you on anything […]’ see what they say […] about that, what – obviously for some reason contraception has gone wrong either because it wasn't used, or they did something wrong with it, or, or it failed them. […] I would then say, ‘Well maybe what we need to look at, assume, so let's say assuming we go ahead with the termination then we need to put a plan in place to try and prevent you getting into this situation again and then look at what the contraceptive options would be from there’. (Consultant 3, female)

For Consultant 3, a woman's capacity to produce an ‘unplanned’ pregnancy (as signified by her request for abortion) illustrates that something has ‘gone wrong’ with contraception, *even in the absence of previous contraceptive use*. Likewise, for GP7, abortion represents automatic evidence of ‘contraceptive failure’. Through such statements, contraceptive control over fertility is positioned as a natural (i.e. normal) female bodily state, which has broken down and can be restored by health professionals – rather than a state which women may, or may not, decide to try to achieve using technological assistance.

However, even as they naturalise contraceptive control over pregnancy, health professionals highlight the socio-material work required to *produce* this naturalisation. This is because they position the healthcare interaction as a site where the possibility that women might make active choices with regards to the use/non-use of contraception/abortion is ever-present. The existence of this alternative form of female subjectivity is highlighted (and negated) in the previous extracts through the explicit description of future contraceptive use as a non-choice: it is something, health professionals emphasise, that patients ‘need to think about’.

The work involved in naturalising contraceptive control over pregnancy becomes particularly clear when health professionals describe interacting with women who return to the clinic to request subsequent abortions:

…if someone […] was on their third termination we would say to them, ‘You know, we can do terminations for you, obviously. But if you're going to repeatedly attend for terminations some of the doctors will not sign the forms and we need to look at your contraception – why has this problem occurred for a third time?’ Or you may find ladies will attend and they say they've never attended before and you look back in the notes and there is a termination sheet in their notes. And I would just say, ‘I see you've attended for a termination before and, you know, has this been a failure’ – so I would say it in the nicest possible way you know for a second one […] I think the worst I've ever seen was […] I think she was maybe on her fourth and we said to her, you know, ‘This is no longer acceptable. And we can't refuse people to have terminations but, you know, this is not just a failure of contraception and […] I'm not sure if the doctor will sign the form, so you have to make sure that your contraception is in place or we can't say that we would see you back here or be able to offer you a termination on another occasion’. So it's said in a very pleasant way, but firm. (Nurse 9, female)

As a woman's number of return visits to the clinic increases, the contraceptive ‘problem’ evidenced by abortion is located ever more precisely as a ‘failure’ of female subjectivity (rather than, for example, a ‘failure’ of technology). This finding replicates that of other studies of abortion work (Joffe, 1986; Lipp, 2010; Luker, 1975; Nicholson, Slade, & Fletcher, 2010), which have long highlighted that women who repeatedly request abortion become demarcated as an irresponsible group of patients by staff. In the context of this paper, however, the analytically interesting issue is the way in which health professionals construct their response to this perceived problem. Specifically, they describe intensifying their efforts to construct female subjects for whom contraception is an automatic state of ‘being’, rather than an active choice. On the one hand, such denials of female reproductive agency have troubling implications for individual patients. On the other, health professionals' descriptions of their struggles to produce particular kinds of female subject also demonstrate that the possibilities of female subjectivity (and correspondingly of contraception/abortion) are multiple.

### Working on/with female fertility

The extracts presented above highlight potential ambiguities in the nature of the contraceptive ‘problem’ which is indicated by abortion. Beyond particular thresholds (which varied between health professionals), a woman's number of previous abortions was described as evidence of a ‘failure’ of female subjectivity. Below these thresholds, however, health professionals talked about, ‘a failure of contraception’ and even the possibility that contraception might ‘fail women’ (see previous quotation from Consultant 3). In other words, they frequently described contraceptive techniques themselves as sites of nonhuman action which shape female fertility.

Ultimately, in the majority of health professionals' accounts, the activities of nonhumans such as contraceptives were positioned as subordinate to human actions. At the same time, however, this positioning was described as an *achievement*, which requires prior knowledge of the myriad ways in which nonhuman entities may act:

…somebody's got pregnant three times on the Pill. Everybody thinks the Pill's the be all and end all because it's one of the most reliable methods of contraception. And - but if somebody's still getting pregnant on the Pill are they not taking it correctly? Is it the best thing for them? What - are they taking other drugs that will interact, have they had antibiotics? […] Are they aware of the extra precautions you need to take, if you have diarrhea and vomiting and things like that? (Nurse 8, female)

Through such accounts, health professionals reveal the complexity of the socio-material work upon which the enactment of abortion as an indicator of a ‘fixable’ problem with contraception depends. In highlighting the possibility that human attempts to control female fertility will be thwarted by the actions of nonhuman entities (for example, contraceptives, other drugs, viruses, etc.) they position the subordination of female fertility to the rationality of ‘the will’ (Ruhl, 2002) as an intrinsically less certain venture than it appears within policy guidance.

### Working on/with clinic protocols

Another form of nonhuman action which featured with great regularity in health professionals' accounts was that of clinic protocols (both electronic and paper-based). This process is evident in the following extract, where the computer systems that monitor patient attendance at follow-up appointments are cited as participants in the process of correcting the contraceptive ‘problem’ that has been signalled by a patient's request for abortion:

…the strategy is usually well, this has happened, now we're going to get it sorted out, but it's really really important that you have effective contraception afterwards – you can become pregnant almost immediately. So let's think about giving you some of the Pill now or arranging for you to have an implant put in your arm or you come back at, you know, within a week and have an IUD put in. So we, a very big focus is trying to prevent repeat abortions in people. […] We have a system where if they have made an appointment their notes will be flagged up if they don't attend, and they get, they will get a letter to say that we'd like to see them, we notice they haven't, they haven't attended, please could they make arrangements to come, it's important that they have a check-up to make sure that everything's fine. (Consultant 1, female)

Likewise, the check-lists used to standardise the information provided to and obtained from patients who request abortion in hospital outpatient clinics were also described as playing a key role:

We have a […] very rigid form to go through […] basically it's fairly straightforward, and we just go through what would be a normal consultation: name, age, how many pregnancies they've had before whether that be live, or terminations, or miscarriages. What they use for contraception. Last period. Smear. We already have an ultrasound to hand. We touch on past medical history, drug history, allergies […]. And then we talk about future contraception. […] They have to leave with some form of identifiable contraception following the termination. (Specialist Registrar 2, male)

The central place accorded to such protocols within health professionals' accounts is significant for two reasons. Firstly, these entities emerge as another important set of nonhuman participants in the enactment of abortion as evidence of a ‘fixable’ problem with contraception. In this sense, they highlight another form of socio-material work (interactions with protocols) that is required to enact contraception/abortion in accordance with policy. Secondly, however, such protocols also provide a *physical link* between the contraception/abortion enacted in the clinic and that enacted by policy. Forms such as the one described by Specialist Registrar 2 do not simply shape the content of health professionals' interactions with patients. Simultaneously (as health professionals are well aware), they allow information to be recorded about the outcomes of these interactions (patient appointments made and/or kept, contraceptives provided, etc.) and thus participate in policy attempts to evaluate the ‘quality’ of abortion services. As such, clinic protocols can be considered as a particularly important technique of ‘co-ordination’ (Mol, 2002, pp. 53–85) through which the contraception/abortion enacted within the clinic become aligned with that enacted through healthcare policy.

### Working on/with heterosexuality

The final form of work that is necessary for health professionals to enact abortion as evidence of a ‘fixable’ problem with contraception is the translation (Mol, 2002, pp. 72–85) of the relationships of heterosexual couples into the bodily health problems of individual women:

They just kind of assume it won't happen to them […] partner doesn't like using condoms or they don't like using condoms, or they got carried away. And you can understand how these things happen but there's still a, a risk of pregnancy with, even if you are carried away, unfortunately, chance or statistics or whatever doesn't make allowances for you being carried away or your partner not liking condoms you still can get pregnant. […] the form does say ‘future contraception decided’ so I would always ask them what they're planning for the […] future […] ‘Well, I'll go back to the condom’, ‘But you didn't use it every time’. It's just trying […] to get them to think about […] the gap between what they think they're doing, and what they're actually doing. (GP3, female)

In this account, heterosexual pleasure (and power) relations are translated into a calculable measurement: the risk that a woman will conceive a pregnancy during heterosexual intercourse. This process transforms an unfixable contraceptive problem (condoms interfering with a heterosexual couples' bodily pleasure) into something (a woman's ‘ignorance’ of the bodily risks which she is taking through her sexual practices) which can be managed by health professionals through their interactions with individual women within the abortion clinic.

A related translation takes place in the following extract:

…at the end my last question would be ‘Now what [do you wanna do] about contraception?’ And if, which they often do say, ‘Well I'm not, never, I'm not planning to have sex again for quite a long time or, you know, just it's not even an issue at the moment, I don't even want to think about it,’ I would probably say, ‘Well I know you feel like this right now but actually you may not feel like this in a few weeks' time and it's, for you it's probably really important that you have good contraception. And so although it doesn't feel right it would be good if we could agree, or you could agree what method you want to use. And even if you're not in a relationship just start it so that you're in control of that and […] you have contraception.’ (Nurse 7, female)

In this account, patients who seek abortion attempt to enact contraception as a technique of fertility control that requires on-going heterosexual activities/relationships. In the face of this, Nurse 7 describes having to engage in particular kinds of interaction with a patient to translate a contraceptive non-problem (the absence of heterosexual activity) into a contraceptive problem (individual women's well-being) that health professionals and women can resolve within the context of the clinic.

A final example of the ways in which health professionals translated heterosexuality in order to enact ‘fixable’ contraceptive problems is provided by instances in which interviewees' explained their promotion of women-controlled methods of contraception:

P: And then at the end when […] you've done the referral I always say, ‘Well […] what are you going to use after?’ […] I always want my ladies to say, ‘Yes I will use this after,’ and I probably wouldn't let […] them get away with, ‘Oh I don't know,’ I would say ‘No, you need to know’.

I: Um, what if someone says ‘I'll use condoms' or, or whatever, is that?

P: I would probably not be very happy with that because I would, I would say to them, ‘That's fine, you won't get any sexually transmitted diseases, but that's for the man and you're the woman, and you'll get stuck with the problem, failure rate is ten per cent, you need something else.’ You may not push them too much further at that point but I wouldn't be happy with that after a termination because something's gone wrong, you know. (GP10, female)

In this account, a problem that is intractable from a healthcare perspective, namely, the gendered distribution of the burdens of pregnancy and childcare is translated into another kind of problem: a woman's ‘ignorance’ of the gendered distribution of responsibility for the *prevention* of these burdens. This second problem, GP10 suggests, can be addressed through education, which includes raising awareness that the burden of women's responsibility includes acceptance of the use of particular contraceptive methods. The fact that such responsibility also includes acceptance of the bodily risks attached to these methods was stated explicitly by several interviewees:

I do find it a bit more difficult in terms of, you know, I don't feel that comfortable with it […] I think it's [*repeated abortion is*] irresponsible […] I suppose I'll talk more about contraception. I don't think it's as bad as it was in [city] you know, when we had lots of women who almost used […] termination as a form of contraception. Especially people like ballet dancers or with, with anorexia or people that just didn't want to, you know, take any kind of Pill, or yeah anything that might affect their weight. (GP6, female)

Similar dismissals of contraceptive side-effects are observed by Hayter (2007) in his analysis of nurse prescribing practices. Such accounts must be viewed in light of the historical redistribution of the bodily burdens of heterosexuality which has accompanied the development of contemporary contraceptive technologies. The second half of the twentieth century witnessed the development and proliferation of hormonal contraceptives focussed on female bodies (e.g. intrauterine devices, oral contraceptives, implants). As feminist scholars have long argued, such methods provide women with more ‘effective’ methods of avoiding the burdens of pregnancy but simultaneously code bodily responsibility for contraception as a ‘feminine’ rather than a ‘masculine’ problem (Luker, 1975; Oudshoorn, 2003).

### Alternative enactments?

I have suggested that health professionals' illustrations of the work that is required to enact abortion as evidence of a ‘fixable’ problem with contraception implicitly demonstrate the ontologically ‘multiple’ nature of both abortion and contraception. Another way in which this multiplicity is rendered visible by the interview data is through the alternative sets of practices through which contraception/abortion was enacted by some health professionals. Many of these alternative practices are exemplified by the following extract from GP8. I quote this interviewee at some length because, in reflecting on the issue of so-called ‘repeat’ abortion, she enacts several contraceptions/abortions which bear little resemblance to those considered in the discussion so far:

P: Well I mean in Eastern Europe it's the main form of fertility control. I'm not saying it's the right form […] it's rather as if, you know, the horse had bolted. But it is definitely a form of individual fertility control. Um it's not encouraged in this country, there's lots of better ways of controlling your fertility. But repeat aborters are, I think, a fascinating group […] You know […] there's such a grade of behaviour about sort of concepts or constructs about health and medical treatment and what not. I mean some women think absolutely nothing about having operation after operation after operation, and the sort of Laura Ashley brigade wouldn't even dream of taking contraceptive Pills or, you know, some women probably think there's nothing wrong with having termination after termination. But I mean it, I think it's sometimes to do with coercive partners. And I have no truck at all with these arrogant gynaecologists who wag their fingers and say, ‘You silly girl’ and things. […] But I think it also depends on women's fertility I mean a lot of women get away with it, whereas some women seem to be exceedingly fertile!

I: When you mentioned coercive partners before was that, were you referring to sex, or to […] coercing women into terminations?

P: Well, could be either really. But when I say um unplanned, unwanted pregnancies, coercive partners who, for one reason or another don't use condoms, for instance. (GP8, female)

Towards the beginning of this extract, abortion is enacted not as evidence of women's ‘failure’ to control their reproductive bodies through contraception but as evidence of an (albeit inadvisable) form of female reproductive agency. Through its connection to healthcare provision more broadly, contraception/abortion then becomes evidence of the contested status of ‘health’ and the varieties of ways in which patients enact this concept through their approaches to medical treatment. Further on, contraception/abortion is suddenly reconfigured again through the refocusing of the conversation onto the fertility of women's bodies. Such fertility is enacted as a state that is unevenly and uncertainly spread across the female population. Through this practice, rather than evidencing a patient's ‘problems’ with contraception, abortion is transformed into an indicator of a patient's *fertility*. Finally, when GP8 emphasises the heterosexual relationships through which pregnancies are produced, she enacts abortion as another kind of indicator: of the constraints which such relationships can impose on the forms of reproductive agency available to women.

## Conclusion

This paper has utilised Scottish health professionals' descriptions of the provision of contraceptive advice during abortion consultations to highlight the socio-material practices upon which the realities of contraception/abortion depend. In illustrating these processes, it has provided new empirical insights concerning the provision of advice about contraception in Scottish abortion practice. It has also demonstrated that conceptual insights from STS (in particular, the work of Mol, 2002) offer a useful framework through which to extend the scope of existing social scientific enquiry concerning the provision of techniques of fertility prevention.

As highlighted in the introduction, literature in this field has tended to position nonhumans as uninvolved in, and unaffected by, human (social) actions. In contrast, the analysis presented here has illustrated how the reality of contraception/abortion depends upon a complex set of human–nonhuman interactions (or socio-material practices). By drawing attention to the complexities of this work, it has rendered visible the ontologically ‘multiple’ (Mol, 2002) nature of these techniques of fertility prevention, thus affording opportunities to consider that the reality of contraception/abortion might ‘be done differently’ (Law, 2008, p. 635).

Whilst emphasising this potential multiplicity, however, the preceding discussion has also highlighted the way in which contraception/abortion realities are *contained* (or, to use Mol's term, ‘co-ordinated’). Specifically, it has demonstrated the extent to which the contraception/abortion reality described by health professionals converges with that enacted by healthcare policy. One reason for this is suggested by the linkages which nonhumans, in the form of clinic protocols, create between the contraception/abortion enacted through policy and that enacted in the clinic. Through healthcare quality targets and written policies, rising abortion rates are enacted as evidence of problems with contraception which health professionals have ‘failed’ to fix. Within the clinic such enactments are ‘made durable’ (Latour, 1991) through referral forms which ask GPs to indicate what future contraception their patients will be using, and through clinic protocols which require women to leave with an ‘identifiable form of contraception’.

The analysis presented here has clear implications for those involved in formulating such policies, because it raises important questions about the forms of healthcare practice which these policies sustain. Specifically, as noted above, in order for health professionals to enact contraception/abortion in ways that accord with policy, they must perform particular kinds of work on female fertility, female subjectivity and heterosexual relationships. This work involves transforming female fertility into a predictable, controllable state, translating heterosexual relationships into a ‘problem’ of individual women's health and – perhaps most significantly – transforming women seeking abortion into subjects who have no agency in relation to techniques of fertility prevention.

Indications of ways in which the contraception/abortion reality might be – and is sometimes - ‘done differently’ emerge from the minority of cases in which health professionals described engaging in alternative socio-material practices. These practices included treating abortion as evidence of an uneven and uncertain distribution of fertility which ‘burdens’ some heterosexual women's bodies more than others, as evidence of the power differentials which routinely characterise heterosexual encounters, and as a form of individual reproductive agency. Through such practices, abortion becomes decoupled from contraception in ways which could arguably be said to ‘multiply’ the forms of subjectivity available to health professionals and their patients.

In drawing attention to these alternative enactments, this analysis is also a situated (Haraway, 1991) intervention, which produces particular kinds of reality through particular kinds of practices. Most notably, it has relied upon a very limited set of data (health professionals' interview accounts) to make arguments about the realities that may be enacted during the provision of contraceptive advice within consultations about abortion. Even if there are good reasons to treat health professionals as ethnographers of the events that have happened to them (Mol, 2002), interviews with this single group of actors clearly provide very restricted insights into the socio-material complexities of healthcare practice. In addition to preventing the observation of human–nonhuman interactions, this form of data makes it difficult to consider the practices which *patients* may engage in to enact themselves as agents during encounters with health professionals (Lowe, 2005; Lupton, 2003). Observation of the dynamics of health professional–patient interactions concerning contraceptive/abortion provision represents one important means through which future research might address this limitation. Perhaps more crucial however, is the development of a better understanding of the ways in which the realities of contraception and abortion are enacted through the practices which Scottish women *and men* engage in, beyond the medicalized context of the clinic.
